# Understanding healthcare providers’ experiences with video recording of patient consultations

**DOI:** 10.1017/S1463423622000214

**Published:** 2022-06-09

**Authors:** Charlotte Gjørup Pedersen, Lea Høj Høstrup, Birgitte Bitsch Gadager, Claus Vinther Nielsen, Thomas Maribo, Louise Sofia Madsen

**Affiliations:** 1 DEFACTUM, Central Denmark Region, Aarhus, Denmark; 2 Department of Public Health, Aarhus University, Aarhus, Denmark; 3 REHPA, The Danish Knowledge Centre for Rehabilitation and Palliative Care, Odense University Hospital, Nyborg, Denmark; 4 Department of Clinical Research, University of Southern Denmark, Odense, Denmark; 5 Department of Clinical Social Medicine and Rehabilitation, Gødstrup Hospital, Herning, Denmark

**Keywords:** focus-group interviews, healthcare providers, patient consultations, video recording

## Abstract

**Aim:**

To understand healthcare providers’ experiences with video recording of patient consultations.

**Background:**

Video recordings have been recognised to be an effective method to evaluate in situ interactions in clinical practice. The video recordings are often conducted by researchers, but active involvement of healthcare providers into the process of recording is evolving. Still, little is known of how video recordings by healthcare providers may influence daily clinical practice and potentials for direct use to guide practice development.

**Methods:**

A qualitative design was used, conducting two focus group interviews including 12 healthcare providers representing eight different healthcare services who provide municipal cardiac rehabilitation. Interpretive description was used as the methodological framework, and symbolic interactionism served as the theoretical lens.

**Findings:**

Three themes were identified reflecting healthcare providers’ experiences with video recording of patient consultations: ‘Concerns of compromising primary work tasks’, ‘Exposing professional and personal skills’ and ‘A new learning dimension’. Overall, the three themes represent the process of video recording own practices attached to patient consultations and the personal investment attached to the video data. Also, how the recordings may provide new insights for practice development in terms of individual and team-based performance in patient consultations.

**Conclusion:**

Video recordings by healthcaref providers may be a useful source to provide information and learning about patient consultation practice to use in research and supervision, keeping in mind their challenges of implementation into daily clinical practice.

## Background

Video recordings have been recognised to be an effective method to evaluate in situ interactions in clinical practice (Asan and Montague, 2014). Over the last years, there has been an increasing use of video recordings (Baumann *et al*., 2020). Yet, the most common public health topics studied using film methods are adolescent health (Baumann *et al*., 2020), studies describing and analysing patient–practitioner interactions (Cahill and Papageorgiou, 2007; Heath *et al*., 2007; Henry and Fetters, 2012; Asan and Montague, 2014) or evaluations of communication competencies (Holmström and Rosenqvist, 2004; Rosengren *et al*., 2005; Iedema *et al*., 2009a; Henry and Fetters, 2012; Barratt and Thomas, 2018). The advantages of video recording are that all or selected situations can be recorded from beginning to end to gain insight into verbal and non-verbal communication, patient behaviour, or clinical practice (Coleman, 2000; Henry and Fetters, 2012; Meeusen and Porter, 2015).

Approaches to video research varies widely, from well-produced documentary interviews to formats where participants take part in the research project (Morse, 1994; Baumann *et al*., 2020; Pink, 2020). Most often video recordings are conducted by researchers (Larsen *et al*., 2002). Given the full complexity of their own work tasks, active involvement of healthcare providers into the process of recording is not common (Carroll *et al*., 2008). However, healthcare providers’ responsibility for video recording their own practice has been recognised to increase their understanding, ownership and collaboration with researchers (Rycroft-Malone *et al*., 2016; Mitchell and Sommer, 2016; Filipe *et al*., 2017). Also, video is used in contemporary forms of medical education to provide clinicians with visual feedback about how they interact with patient actors to improve their clinical skills and communication (Carroll *et al*., 2008). The use of video recordings in supervision hold potential to influence healthcare providers’ self-perception, increase self-analysis and enable supervisors to evaluate clinical practices more accurately (Huhra *et al*., 2008; Dohms *et al*., 2020).

Still, little is known of how video recordings by healthcare providers may influence daily clinical practice and potentials for direct use to guide practice development. In this study, we focus on the experiences and circumstances of recording the videos when produced by the healthcare providers themselves. Given the rise in user-friendly technology that makes such methods even more accessible (Schwab-Cartas and Mitchell, 2014; Miller Scarnato, 2017), this is an opportune time to gain a further understanding of healthcare providers’ experiences of video recording (Baumann *et al*., 2020). This study is based on cardiac rehabilitation and patient consultations at primary healthcare centres; settings with no tradition of systematic evaluation of the content in these consultations. It is therefore relevant to investigate whether it is possible to conduct and use video recordings as real-life data source to improve daily clinical practice at primary healthcare centres.

The aim of this study was to understand healthcare providers’ experiences with video recording of patient consultations.

## Method

### Context of the study

This study is based on healthcare providers’ experiences with video recording of patient consultations, and these recordings were part of a cluster-controlled trial, which is referred to as main project.

The main project examines if a given specific evidence-based patient education strategy used in cardiac rehabilitation would result in higher program completion rate and better self-management compared to another patient education strategy (Lynggaard *et al*., 2014; Varming *et al*., 2015). Healthcare providers from eight primary healthcare centres video recorded the initial patient consultation based on the motivational interview (lasting approx. 1 hour) in a cardiac rehabilitation programme. The healthcare providers were introduced to the research aim and methods at project start. The healthcare providers were responsible for logistical processes related to video recording in daily clinical practice, setting up the video equipment (a tablet facing the healthcare provider and recording only the patients’ voices) and using an app to record and upload the video to a project server. Subsequently, the videos were analysed by external researchers with expertise in the motivational interview to evaluate the use of this theory in the patient consultation. The healthcare providers had no experience in video recording of their own practice; therefore, a workshop in video recording and technical support (hotline) was essential and provided from the research team. The research team ensured that written informed consent was conducted from all healthcare providers prior to participating in the main project and video recording, which were performed in accordance with General Data Protection Regulation (GDPR). Accordingly, the healthcare providers were responsible conducting written informed consent from all patients before starting the video recording of the patient consultation, although not displaying the identity of the patient. Video data were collected from 1 August 2018 to 31 July 2019. A total of 335 videos were recorded. Subsequently, healthcare providers participated in individual- or group-based supervision using their own videos for feedback. The supervision was led by external experienced psychologists.

#### Methodology

Interpretive description was the chosen research methodology, to generate qualitative and practice-relevant knowledge inductively in the field (Thorne, 2016). Interpretive description provided a coherent conceptual description of the data material, on the basis of patterns and relations within data (Thorne, 2016). To generate empirical and theoretically grounded knowledge, interpretive description was accompanied by symbolic interactionism as the theoretical framework (Blumer, 1986). Symbolic interactionism is based on the assumption that people act on the basis of personal meaning, which arises from, and is modified by, processes of social interaction with others (Blumer, 1986). In this study, interpretive description and symbolic interactionism were used to enhance on the complex social actions and sense-making attached to the health providers’ experiences of recording videos (Blumer, 1986; Oliver, 2012).

#### Participants

In total, 12 healthcare providers were included based on a purposive sample strategy (Thorne, 2016). In total, all 19 healthcare providers from the eight primary healthcare centres who had participated in recording the videos were invited, of which seven declined due to high work pressure in the clinic. The participants in this study were recruited from the main project and included health care providers who had performed the video recordings. Participant characteristics are shown in Table 1. The number of participants differed across the eight centres: One health centre included four participants, two health centres included two participants and four health centres included one participant. The participants in the two focus-group interviews were divided based on a pragmatic approach regards the geographical distance between the primary healthcare centres, giving the shortest travel time for the participants and thus contained mixed professions.

**Table 1.** Participants characteristics

Participants:	*N*=12
Gender - Men - Women	*n*=1 *n*=11
Professions:	
- Physiotherapists - Nurses - Dietitian	*n*=6 *n*=5 *n*=1
Professional experience in healthcare	Mean 18.4 years
Worked with cardiac rehabilitation	Mean 3.7 years

#### Data generation

In-person focus group interviews were chosen to create an interactive context for shared discussions to understand the joint perspective among the healthcare providers (Krueger and Casey, 2014). A semi-structured interview guide was used (cf. Appendix 1), with questions developed on the basis of existing evidence regarding video recordings and healthcare providers’ experiences with video recording, like for instance the patient–healthcare provider relationship or the extensive logistics involved (Heath *et al*., 2007; Asan and Montague, 2014; Parry *et al*., 2016; Pino *et al*., 2017). The participants discussed open-ended questions, for instance: ‘*Can you tell me about your initial thoughts on participating in video recording consultations?*’ and ‘*How did you feel about being video-recorded during consultations?*’. Second author moderated the focus-group interviews and the last author participated as assistant moderator, posing follow-up questions (Krueger and Casey, 2014). Each focus group interview lasted two hours and was audio-recorded.

#### Data analysis

Data were analysed by drawing on the interpretative description methodology (Thorne *et al*., 1997), applying four iterative analytic steps moving from pieces into patterns through inductive interpretive processes (Handberg *et al*., 2015).

1. The focus-group interviews were transcribed verbatim and read by the author team who initially discussed first impressions and content of the material. Data were uploaded to NVivo^TM^. The second and the last author re-read and coded the data material based on an inductive analytic approach, creating the codes from the empirical data material.
2. Initial themes and general patterns were identified. Going back and forth to the raw data and using the study aim and symbolic interactionism (Blumer, 1986) as an interpretive lens, the relationships developing in the data were critically discussed with the author team.
3. Categorical themes and relationships between them were identified. This stage included a critical appraisal of relationships within the data and relevant thematic options – moving from a descriptive analysis to an interpretive analysis through condensation of the material to capture the essence of the data – which yielded the categorical themes, representing the complex social actions and sense-making attached to the health providers’ experiences of recording videos.
4. Memos of each theme – representing provisional understandings and meanings with representative citations – were conducted and co-authored by the first, second and last authors and discussed with the author team. At this stage, the data in Danish language were transformed into memos in English, and citations were directly translated into English with professional assistance.
5. Main messages were extracted based on key insights within data. Illustrative quotes were provided, translated from Danish language. The presented quotes are without identification in order to anonymise the few participants.

## Results

Three themes were identified reflecting healthcare providers’ experiences with video recording of patient consultations: ‘Concerns of compromising primary work tasks’, ‘Exposing professional and personal skills’ and ‘A new learning dimension’. Overall, the three themes represent the process and logistics attached to healthcare providers’ video recordings of their own patient consultations and the personal investment attached to the video data. The concerns and challenges involved with video recording seemed to overall be offset by providing new insights for practice development in terms of individual and team-based performance connected to patient consultations.

## Concerns of compromising primary work tasks

Recording videos of patient consultations were referred to as a resource-demanding task by the healthcare providers, which caused concerns of compromising their primary work tasks. These concerns revolved around the workload of adding an extra task to daily clinical practice and whether it would impact the relation to the patient during consultations. A participant expressed concerns about adding video recordings as an extra element to the patient consultation.

*A lot is going on in this kind of conversation [in the initial patient consultation]. And the video recording was a new element, which we had to incorporate, but did not really have our hearts in… especially not in the beginning. Because it [the video] comes from outside. And I already thought that there were too many things going on in the consultations…and then, in comes this extra…extra ‘thing’. And how much time will this take? Will it distract our work and so on?*

The citation touches upon concerns about core elements essential to the initial patient consultation as described by the healthcare providers: to establish a relationship with the patient, inform about the programme, clarify the patient’s needs and expectations of rehabilitation and goal setting for the intervention. The healthcare providers seemed to have a pre-understanding that the video format would conflict with their work of creating a confidential atmosphere during consultations. A participant shared initial concerns of how video recording could possibly affect the personal investment into the patient consultation.

*I was skeptical about this video recording… video recording myself … and the conversation I had with the patient… that it is a personal matter and when setting up a screen [the tablet] I feared that it did something… it was the screen I was skeptical of in the beginning.*

This citation indicates a general concern about the video recording causing limitations to establishment of a patient-healthcare provider relationship. An initial skepticism towards participating in recording videos seemed to be common among the healthcare providers, with some choosing not to take part in the process. In these situations, the manager appeared to have a central role to play supporting the healthcare providers who chose to take part in video recording by creating a safe environment to reflect and discuss any concerns and to find the best possible solutions in clinical practice.

In the start-up phase of recording videos, practical and technical issues were experienced such as finding a quiet room with reliable internet connection, setting up the video equipment and tablets that inexplicably stopped recordings during the conversation. Further, tasks on recruiting patients and obtaining written informed consent were explained to cause organisational challenges.

It was, therefore, important for the participants that the management acknowledged that video recording demanded additional resources to not compromise the primary work tasks. Only one health centre allocated more time for the new tasks associated to video recording, which seemed to decrease distress among healthcare providers. The healthcare providers also explained that they felt very self-conscious in their first video recordings. Nevertheless, the participants explained how the video recordings gradually became an integrated part of and adjusted into daily clinical practice. Within the first weeks the healthcare providers became more familiar with the task and added their own procedures and techniques to perform video recordings of patient consultations. For instance, several healthcare providers reported that they modified the visible presence of the tablet and placed it on a chair beside the consultation desk. Others placed a piece of paper in front of the tablet screen to avoid seeing themselves while recording. Furthermore, the healthcare providers developed what they explained as a patient-first approach. A participant reflected upon the importance of not letting the recordings distract the patient consultations:

*It was more important to embrace the person sitting in front of you, instead of fumbling half an hour with the IT. I think that we were all good at saying ‘never mind’, if somebody came out frustrated after a patient consultation because the recording did not work. It was just a video recording. After all, the patient is more important.*

The citation illustrate how healthcare providers’ initial concerns of compromising primary work tasks seemed to become manageable to them. The healthcare providers expressed to experience their motivation and ownership of the videos to increase concurrent to their enhanced engagement and personal investment in recording the videos.

## Exposing professional and personal skills

During the process of recording videos, the healthcare providers explained to become aware that the video recordings not only captured their ways of practice and skills as a professional, but also exposed their individual personal skills. The healthcare providers expressed the significance of investing both professional and personal skills into the interaction with the patients during consultations. For instance, a participant explained how the experience connected to video recording of a patient consultation differs from exercising:

*The reason why it [video recording] is intimidating, at least for me, is that this work [patient consultation] demands that I show my personality. It is not just about a professional skill – if I can do a squat properly. This [a patient consultation] is more than doing an exercise. This is about how I act as a person.*

The citation show how the video recordings could be perceived as exposing, because the consultations contained aspects beyond their professional competencies, which were to be evaluated by external researchers, unknown to them. The participants explained how they experienced being recorded on video to be a vulnerable position, by the thought of their personal skills potentially being an object for research. The healthcare providers shared to think about e.g. ‘what do they think of me?’, or ‘are they analyzing me or the method I use?’. Therefore, the healthcare providers gradually became much curious and reflected critically upon the research process. A participant explained how they in the team had experienced more questions to surface once the videos had been recorded:

*Another concern, I think we had, was about…well, now that we have recorded these videos; ‘then who in the big, wide world, is sitting and watching these videos? … We knew they [the video recordings] should be analyzed and looked through. But still, what parameters are looked upon, and who is watching, and how many are sitting…five, ten people… looking at me, having this conversation [patient consultation]. And what are they keeping an eye on about my personality, apart from the topics of the project?*

The citation illustrates the complexity inherent to videos as research data, as healthcare providers felt their personal skills were part of the external research evaluation. After having engaged in recording the videos of patient consultations the health providers experienced to no longer be in control of the video recordings and how they were interpreted. Although the purpose and methods of the main project and video recordings was presented to the healthcare providers prior to project start, some participants shared how they had experienced concerns of being personally evaluated without knowing the scale. To address the ambiguities connected to video recordings exposing professional and personal skills, the health providers discussed different ideas. For instance, the potentials of a closer collaboration with researchers, through involving the healthcare providers’ practice insights and tacit knowledge into the research analyses was suggested. Further the healthcare providers shared much curiosity regards the research analysis of the video recordings, wanting to be involved and gain feedback from their ‘expert’ point of view as well, to benefit from direct learning from a research perspective. Nevertheless, despite ambiguities of exposing professional and personal skills, the healthcare providers found the videos to provide a new learning approach for improvements in practice when used in a supervision setting.

## A new learning dimension

The video recordings of patient consultations appeared to add a new learning dimension to the work of health providers, by providing an avenue for looking at daily clinical practices and routines from outside. The task of producing video recordings of patient consultations forced the health providers to reflect upon own practices, which seemed to induce individual learning experiences. Besides the individual learning connected to recording own videos for research purposes, the video material was used in team supervision making team learning possible. A participant critically reflected upon the learning opportunities in sharing unfiltered material with colleagues.

*I think, that the video is a new dimension, which I thought was good to try, as a part of supervision. There is something about videos, which is revealing, but in a good way, I think. It is not meant in a negative sense. We do know that we can always join in on each other’s consultations, but we never do it. This [using the video recordings] does something else. It presents things more direct…something more…there are no layers on top. There are no assessments. It is kind of raw. Situations appear more naked – unfiltered.*

The citation illustrates the learning opportunities of using video recordings to share experiences for joint discussion on how to improve patient consultations. The healthcare providers expressed how they were dependent on professional guidance to create a ‘safe space’ for sharing vulnerable situations with colleagues and to facilitate the learning process. A participant explained how the recorded patient consultations necessarily reflected a natural variation in performance.

*Some days are off days! So…I may have said it when recording ‘delete that s…’ … when I knew the performance would show a bad result if they [external researchers] used a motivational conversation scanner… the poor patient had just been loaded with information, because I was tired. Other times, I think best conversation ever… which is a pat on the shoulder!*

This citation indicates that video recordings may give the healthcare providers the opportunity to gain insights in one’s strengths and weaknesses. Using video recording for supervision seemed to inspire discussions about how to also improve the team culture. For instance, to give constructive feedback, to be vulnerable, to gain insight into diversity in performance and to have scheduled meetings for working systematically with improving the local cardiac rehabilitation programme. A participant explained how the video recordings enabled new dimensions into daily practice.

*I think there is great learning…or potentially great learning in watching oneself from the outside. How do you actually know…we all have an idea about how we think we act, and how we think the world perceives us, and how we want the world to perceive us. But here, we are given the opportunity to get a view on the matter/case, so to speak. So, I think that this has been really valuable, to me. And really exciting to see my colleagues’ video recordings in relation to how differently we approach the matter/case, and what we can learn from one another.*

The citation supports that general experience among the healthcare providers retrospectively were, that the overall learning benefits from recording the videos and participating in supervision compensated for the extra effort and concerns connected to producing the videos.

## Discussion

The results of this study add to the understanding of healthcare providers’ experiences with video recording of patient consultations. Conducting video recordings of patient consultations initially caused concerns among the healthcare providers of compromising daily clinical practices at the cardiac rehabilitation centres. Management support was important to succeed with video recordings. Furthermore, collaboration between healthcare providers and researchers appear as central to prevent healthcare providers’ distress of exposing professional and personal skills and support learning. Supervision using video recordings has potentials to add new learning dimensions and assist the process of mobilising knowledge into daily clinical practice and motivated overcoming the challenges of video recording.

Confidence in using the video equipment and management support are important components to succeed conducting video recordings. In line with other studies, the healthcare providers found video recordings technically- and logistically challenging and time-consuming (Halimaa, 2001; Henry and Fetters, 2012). Therefore, it was crucial that all healthcare providers had participated in the workshop on video recording and had access to technical support (hotline). For success in video recording, healthcare providers must be confident in using the video equipment, as inadequate training has been cited as a barrier to implement new initiatives (Mathieson *et al*., 2019). Further, our findings support the importance of management support and allocation of organisational resources to conduct video recordings. In addition, facilitators that make healthcare providers adopt new tasks should be taken into account – these have been stated as saving clinical time, increasing cost-effectiveness, to improve nurse–patient relations and patient care and to support meeting organisational goals (Mathieson *et al*., 2019).

Besides the practicalities involved in video recording, the healthcare providers were initially concerned about whether the recordings would conflict with their work of creating a confidential atmosphere during consultations. In the literature, video recordings have been demonstrated to have little or no effect on patients’ and healthcare providers’ behaviour (Pringle and Stewart-Evans, 1990; Coleman, 2000). While ‘social desirability bias’ is raised as a limitation, involving that participants may be influenced by knowing their videos could be viewed by others; acting in accordance with what is expected of them or in compliance with how they want to be perceived (Catalani *et al*., 2011; Lundström *et al*., 2012). Other studies indicate that performance anxiety connected to recording videos for evaluation may influence healthcare providers’ ways of acting (Huhra *et al*., 2008; Stokes and Cummins, 2013). Although the healthcare providers in this study expressed to be able to continue ‘practice as usual’ after the first recordings, using video recordings in research and supervision should be done with critical consideration on potential biases at stake. In that context, it would be relevant to include the patients’ perspectives, which was not included in our study.

Continually dialogue and deliberation are important when researchers and healthcare providers collaborate on video recordings. Healthcare providers seemed concerned about their personal skills as research objective and being evaluated without knowing on which scale. This result may relate to the healthcare providers’ deficient knowledge of the project evaluation and ethics. The healthcare provider’s concerns may also indicate an insufficient level of information from the research team to the healthcare providers. The literature indicates that healthcare providers seem more likely to engage with a research project if they understand the benefits of the research and if it was personally meaningful and helpful to them (Williams *et al*., 2020). Literature suggests that researchers could benefit from training on how to present their work in order to facilitate healthcare providers and researcher collaborations (Williams *et al*., 2020). To address ethical issues of insecurities arising and to ensure continued engagement, using layers of consent has been suggested as a strategy where participants can choose between different levels of consent (Sagan, 2012). Furthermore, it is recommended that the consent is continuously discussed with the participants throughout the process as feelings regards participation may change over time (Brinkmann and Tanggaard, 2020). Another aspect to consider are the increasing body research that point towards benefits of involving study participants actively into mores stages of the research process (Brett *et al*., 2014). For instance, although a different method, a review of photovoice studies found that the strongest studies were those incorporating participants throughout all stages of the study (Catalani and Minkler, 2010). Considering the findings of health professionals experiencing ethical concerns exposing personal skills through videos, involvement throughout all research stages may have potentials as a preventive strategy.

The new learning dimension experiences through the reflective processes accompanying the recording of videos and through supervision seemed to be an important motivating factor to the healthcare providers because of the potential for personal and team-based improvements. Video-based feedback has been recognised to assist healthcare providers in tapping into the visual and auditory patient cues present in a consultation that are not available through text-based learning (Kamin *et al*., 1999), where showing becomes a way of saying the unsayable (MacDougall, 2006). In line with other studies, the videos made the healthcare providers aware of taken-for-granted acts and ‘tacit knowledge’, which have been argued to be fundamental resources of knowledge in improving practice (Rolfe, 1998; Cheater, 2003; Meyer, 2003; Iedema *et al*., 2009b). Further, video recordings have been recognised to enable healthcare providers to critically engage with their own practice norms (Carroll *et al*., 2008; Carroll, 2009; Iedema *et al*., 2009b; Crenshaw, 2012). Considering the overall findings of this study, the general experience among the healthcare providers retrospectively was that the learning benefits from recording the videos and participating in supervision compensated for the extra effort and concerns connected to producing the videos.

### Methodological considerations

The credibility of the study was sought enhanced through applying a systematic approach from planning and design, conducting the interviews and throughout the analysis by creating a study protocol before initiating the study (Thorne, 2016). Transparency was sought through all stages by continuous adjustment of the study protocol, reviewed by the research team. Moreover, the transcripts were read by and discussed in the whole research team, which enhanced trustworthiness of the results (Thorne, 2016). The author group represented different professional backgrounds (anthropology, nursing, medicine and physiotherapy) promoting critical discussion and helping to prevent the personal or disciplinary biases of a single researcher from excessively influencing the results. To prevent pre-understandings from the main project to interfere with the data generation of the present study, the focus-group interviews were conducted by the second and last author who are external researchers with no previous knowledge of the main project. The residual of the research team was involved in the main project and their insights and relations were relevant for accessing the field, recruitment and qualifying data analysis and dissemination.

Our sample size may be considered a limitation. Although a bigger sample was sought for, it was not possible for all invited healthcare providers to participate due to high work pressure in the clinic, which seem a general weakness in clinical research. However, using Malterud *et al*. concept of information power our sample size was considered in alignment with the narrow research question and the specific nature of the phenomenon (Malterud *et al*., 2016). Another limitation is that few primary healthcare services were overrepresented in form of number of participants in the interviews, thus their experiences may be strongly represented in our data. For example, some healthcare centres were more challenged with recruitment as well as technical difficulties in video recording than other centres that may have impacted on the results.

The choice of focus group interviews showed to be appropriate for exploring, analysing and describing the research question. However, Thorne suggests triangulation of data sources as a means to increase credibility by which analytical standards and conclusions can be generated from the synthesised data (Thorne, 2016). Thus, adding observations of healthcare providers’ process of making video recordings may have provided different insights to the results of this study, but may have interfered with the patient consultation. Further, the patients represent a relevant perspective, which was not included in our study, based on ethical reasoning. The persons in cardiac rehabilitation may be in a vulnerable position experiencing severe loss in their physical, mental or social functioning and thus we sought to limit the impressions for the patients during the consultation.

At an institutional level, ethical considerations exist as well, regards the working environment for the healthcare providers. To some healthcare providers’ video recording one’s own performance in a patient consultation may be uncomfortable and cause distress and may ultimately influence job satisfaction. Therefore, an important implication for practice is management support and creating a safe environment for the healthcare providers to reflect and discuss any concerns, create the best possible solutions in practice and also support the choice of declining to participate in the task of video recording. The transferability of the results is considered relevant in health services other than cardiac rehabilitation, as similar experiences may appear in other contexts involving video recording of patient consultations.

## Conclusion

Video recordings by healthcare providers may be a useful source to provide information and learning about patient consultation practice to use in research and supervision, keeping in mind their challenges of implementation into daily clinical practice. Management support was important to succeed with video recordings. Using video recordings as research data demands a high degree of involvement and clarification during the whole research process to create a trustful relation and a safe work environment for all involved parties. Supervision using self-produced videos was a motivation factor for video recording and overcome the challenges of recording. Supervision using video recordings may promote learning based on specific practice experiences and situations form healthcare providers own daily practice.

## Appendix 1: Interview-guide. The interview-guide is translated from Danish language to English

**Table:**

First, I will ask you to think back to when you were asked if you would participate in recording videos of your patient consultations…	
1	*Can you tell, what was your initial considerations in relation to video recording patient consultations?*
2	*Did you have any joint discussions with your colleagues in the heart team before you agreed to participate in the project? (What did you talk about?)*
Now I will ask you to think back to after you had agreed to participate and were preparing for the first consultations to be recorded…	
3	Before starting the actual recording of a consultation, you had some preparation to do. How did you experience getting ready for recording the videos?
4	Can you tell about you experiences of asking the patients to participate in the video recordings?
5	How did you experience having the consultations recorded?
6	Did it affect your or the patients behavior that the consultation was recorded? (In which way?)
7	Did the recording of the consultation affect the content of the conversation? (In which way?)
8	Did your experience of being recorded change over time – from first to last recording?
Now I want to ask about the supervision that you received afterwards … We are aware that your supervision has been organised differently, with some in teams and others individually…	
9	Can you tell about your experience with supervision based on your own video recordings from patient consultations?
10	Prior to the supervision, you should choose a video recording (and some might have to choose more) to speak from. What was the basis for your choice of recordings?
In summary…	
11	What are your overall experience from participating in the video recording of patient consultations?
12	If we were to do a similar project with video recording again, what should we do differently?
13	Is there anything I forgot to ask about, or something else you would like to tell in relation to participating in the project?
